# Effectiveness of premedication for hypersensitivity reactions to radiocontrast media in one tertiary hospital

**DOI:** 10.1186/2045-7022-4-S3-P74

**Published:** 2014-07-18

**Authors:** JaeWoo Jung, Hye-Ryun Kang, Byoung-Whui Choi

**Affiliations:** 1 Chung-Ang University Hospital, Korea, Republic of; 2 Seoul National University College of Medicine, Department of Internal Medicine, Korea, Republic of; 3 Chung-Ang University Hospital, Internal medicine, Korea, Republic of

## Introduction

For patients with a known history of hypersensitivity reactions to radiocontrast media (RCM), premedication with antihistamines and corticosteroids before receiving RCM is recommended. It has been shown that such premedication decreases the frequency of mild and aggregate allergic like reactions. However, premedication does not prevent all subsequent reactions.

## Objectives

We investigate the effectiveness of premedication for hypersensitivity reactions to radiocontrast media (RCM) in one tertiary hospital.

## Methods

We reviewed electronic medical records of all the patients who had been prescribed premedication with systemic steroid or antihistamine for hypersensitivity reactions to RCM from January 2009 to January 2012 in Seoul National University Hospital. Data of previous hypersensitivity reactions and reactions after re-exposure to RCM with premedication was extracted.

## Results

A total of 401 patients with a history of hypersensitivity reaction to RCM and were premedicated at re-exposure to RCM. Male was 177 (44.1%) and mean age was 55.83¡¾11.92 years. Most common hypersensitivity symptom was urticaria (259, 64.6%), followed by itching (36.7%), rash (21.4%) and angioedema (13.2%). Hypotension was reported in 9 (2.2%) and syncope 5 (1.2%). Systemic steroid was prescribed in 143 patients (35.4%). After premedication, 139 patients (77.7%) had no hypersensitivity reactions to re-exposed RCM. Incidence of symptom after premedication such as uriticaria (12.4%), itching (5.6%), rash (5.1%), angioedema (2.2%) were significantly decreased compared with previous incidence (P<0.001). However, incidence of hypotension (1.1%) and syncope (0.6%) were no significant different with previous incidence (P>0.05)

## Conclusion

The use of corticosteroid or antihistamine before administration of RCM may diminish the overall prevalence of reactions. Randomized controlled studies of the effects of pretreatment in patients with hypersensitivity reaction to RCM were needed.

